# Influencers and preference predictors of HPV vaccine uptake among US male and female young adult college students

**DOI:** 10.1016/j.pvr.2018.03.007

**Published:** 2018-03-23

**Authors:** A. Scott LaJoie, Jelani C. Kerr, Richard D. Clover, Diane M. Harper

**Affiliations:** a University of Louisville, School of Public Health and Information Sciences, Department of Health Promotion & Behavioral Sciences, 485 East Gray Street, Louisville, KY 40202, United States; b University of Michigan, School of Medicine, Department of Family Medicine, 1018 Fuller Street, Ann Arbor, MI 48105, United States

**Keywords:** Knowledge, HPV vaccine, Young adults, Preferences, Safety

## Abstract

**Objective:**

The purpose of the study was to assess the knowledge, attitudes and beliefs of male and female college students in Kentucky about HPV associated diseases and vaccines, and to determine which parameters predicted self-reported uptake of HPV vaccination.

**Materials and methods:**

A self-selected cross-sectional sample of college students completed an evidence-based online survey.

**Results:**

Of approximately 1200 potential respondents, 585 completed the survey. The average age was 20.6 (SD 3.15) and 78% were female; 84% of the population had had one or more sexual partners. Concern for HPV vaccine safety and potential need for boosters did not significantly deter vaccine uptake. Likewise, knowledge about HPV associated cancers was not predictive of vaccine uptake. On the other hand, parental influence for vaccination was a strong predictor for vaccine uptake (aOR = 5.32, 2.71–13.03), and free vaccine nearly doubled the likelihood of being vaccinated (aOR 1.90, 1.05–3.41). In addition, the strong preference for the respondent's partner to be HPV vaccinated predicted vaccine uptake (aOR = 4.04, 95% CI: 2.31–7.05), but the lack of preference for partner vaccination predicted an unvaccinated self (aOR = 0.50, 0.27–0.93).

**Conclusions:**

HPV vaccination has been successful in young adult college students in Kentucky. Young adults prefer their partners to be HPV vaccinated regardless of whether they themselves are vaccinated. Parental influence and free vaccine were positive predictors for vaccine uptake in this population.

## Highlights

• Young adults have a very strong preference for their partner to be HPV vaccinated.
• Doctors do not influence young college adults’ decisions to be HPV vaccinated.
• Parents are a significant influencer for young adult HPV vaccine uptake.
• Young adults realize need for cervical cancer screening regardless of vaccine.

## 1 Introduction

The advisory committee on immunization practices (ACIP), a body delegated by the Centers for Disease Control and Prevention (CDC) in the US, has recommended human papillomavirus (HPV) vaccination for females 11–26 years old since 2006, and males 13–21 years old since 2011 [1]. Males 22–26 years old may also choose to be vaccinated. In 2013, the National Health Interview Survey (NHIS) started collecting the age at which the first dose of HPV vaccination was received, seven years after approval for females and two years after approval for males [2]. The first report in 2014 indicated that 40% of females and 8% of males in the US, 19–26 years old, reported starting the series.

Traditional demographic descriptors from prior research in collegians are female gender, single marital status, non-Hispanic white race and younger age predicting an acceptance of HPV vaccination [3], [4]. Female college students in Taiwan have a higher chance of HPV vaccination if there has been a family history of a gynecologic cancer, and if the woman feels empowered about her health care [5]. Male college students in the US, ages 18–21 year old, are more likely to be vaccinated than the 22–26 year olds, and less likely if they are living with their partner [3]. These predictors of young adult HPV vaccine uptake, while descriptive [4], [5], [6], [7], have not been explored more thoroughly, such as understanding expectations of a prospective sex partner. Limited inferential studies have evaluated the impact of knowledge parameters, cost of vaccine and concepts of self-efficacy and decision making on actual HPV vaccine uptake [4].

The purpose of this study is to evaluate, as predictors of HPV vaccine uptake, knowledge levels of young college-attending adults about HPV associated cancers, HPV vaccines, as well as attitudes, beliefs and behaviors regarding HPV vaccination. Additionally, the study initiates an assessment of the perceived influence the student felt he or she had in the vaccination decision.

## 2 Methods

### 2.1 Setting

Participants were recruited from a metropolitan research university in Jefferson County, Kentucky, with an undergraduate enrollment of more than 16,000 students. Students are largely in-state residents (74%) with roughly equal numbers of students from urban and rural areas of the state. African Americans make up 10.2% of enrollment with an additional 12.7% of students classified as other minorities.

Undergraduate students enrolled in an introductory psychology course were eligible to participate in the study. Over the spring and fall semesters, approximately 1200 were eligible to enroll in the study via the department's research participation system, Sona Systems (Estonia). The research participation system, which lists several other studies from which the student could choose, described the current study, indicated the research credit hours (0.5 h) that could be earned, and redirected participants to the survey. The online survey was created and hosted on Qualtrics (Provo, UT) and could be accessed only through the Sona System. Participants could complete the survey on a computer or mobile device.

### 2.2 Survey

Prior knowledge surveys about HPV vaccines, particularly to parents of children, do not have validation statistics associated with them, but rely on verifiable scientific knowledge for accuracy. Our research group developed a similar survey with input from medical and public health researchers of HPV associated cancers, HPV vaccines, sexually transmitted infections, shared decision making, and behavioral health/health promotion. We validated the questions with a process that involved an extensive review of the literature, a pilot test for face validity, and a Delphi method for content validity [8]. The initial draft of the survey was created by two authors (ASL and JCK) who have experience in survey research and who had conducted the preliminary literature reviews. The senior authors (DMH and RDC), with expertise in HPV, HPV vaccination and national vaccination guideline development, independently reviewed and revised the survey. The revised survey was circulated among a small group of undergraduate students who were representative of the population from which the final sample was drawn. We queried survey clarity and understandability through informal cognitive interviews of the small student group.

The final survey consisted of 30-items in four sections. The demographics section assessed race, ethnicity, sexual orientation, past sexually-transmitted infections (STIs) and number of lifetime sexual partners. Assessment of knowledge of HPV and HPV vaccination included responses to whether HPV leads to 12 health outcomes; knowledge of the vaccine's effectiveness, duration of immunization, and the need for continued cervical cancer screening. Assessment of attitudes included the importance of the protecting oneself versus protecting one's partner against STIs, perceptions of the safety of HPV vaccination and other vaccines, willingness to get a booster shot(s) after the initial HPV vaccination series, and desire for sexual partners to be vaccinated against HPV. If not a dichotomous answer choice, the responses were either 5 point scales (very negative, negative, undecided/neutral, positive, very positive) with an additional prefer not to answer option due to the sensitive nature of many of the questions; or an estimate of importance between 0 and 100 (zero being not at all important, and 100 extremely important). Assessment of behaviors included HPV vaccine uptake (0–3 doses of either Gardasil™ or Cervarix™), discussion with partners about their vaccine status, use of condoms, and perceived involvement in the decision to be vaccinated. In addition, items assessed the perceived lifetime likelihood of becoming infected with HPV and/or developing an HPV-linked cancer on a scale from 0 to 100 (0 being never, 100 being certainly).

### 2.3 Statistics

Data were analyzed using SPSS V24 (Chicago, IL) and Dell Statistica v13 (Tulsa, OK). Extensive data cleaning methods, done by two of the authors, included removal of non-respondents, assessment for out-of-range values, unreasonably short (less than 2 min) response duration and visual evidence of patterned responding (e.g., non-varied responses to consecutive items, in the face of interspersed reverse coding). Descriptive statistics, non-parametric statistics (chi-square) and binomial logistic regression were used to establish significant differences. Multivariate logistic regression provided adjusted odds ratios to predict vaccine uptake.

## 3 Results

Of the 645 surveys initiated, 585 completed more than 95% of the questions. The majority of the respondents were female (78%), white (74%), and heterosexual (90%), who had 1–2 lifetime sexual partners (36%). Of the 84% of respondents who had reported having had sex, 76% said their partners were of the opposite sex.

Table 1 describes the distribution and characteristics of respondents who had no vaccination or at least one dose of an HPV vaccine. Most commonly vaccinated were white heterosexual females. Of the 585 respondents, 46% had three doses of either Gardasil or Cervarix, and 56% had at least one dose of either HPV vaccine. More subjects with 6–10 sexual partners were vaccinated.

**Table 1:** Population descriptors by HPV vaccination status.

	**Total population**	**No HPV vaccine**	**At least one dose**
		mean (SD)	mean (SD)
***Age, yrs	20.6 (3.2)	21.6 (3.9)	20 (2.3)
Gender*		n (%)	n (%)
***Male	122 (22)	88 (39)	34 (10)
***Female	432 (78)	139 (61)	293 (90)
Race/ethnicity			
White	451 (81)	183 (81)	268 (82)
Black	64 (12)	20 (9)	44 (13)
Hispanic	3 (1)	1 (0)	2 (1)
***Asian	35 (6)	22 (10)	13 (4)
Other	1 (0)	0 (0)	1 (0)
Sexual orientation			
Heterosexual	495 (90)	200 (89)	295 (90)
Gay/Lesbian	13 (2)	8 (4)	5 (2)
Bisexual	36 (7)	15 (7)	21 (6)
Questioning	9 (2)	2 (1)	7 (2)
Gender of sexual partner			
***Men only	329 (59)	98 (43)	231 (70)
***Women only	95 (17)	68 (30)	27 (8)
Both men and women	44 (8)	22 (10)	22 (7)
Virgin	88 (16)	38 (17)	50 (15)
Number of lifetime sexual partners			
None	90 (16)	39 (17)	51 (16)
1–2	196 (36)	84 (37)	112 (35)
3–5	127 (23)	49 (22)	78 (24)
*6–10	75 (14)	23 (10)	52 (16)
>10	59 (11)	30 (13)	29 (9)
Past history of STIs, including HPV	41 (7)	13 (6)	28 (9)

Of those who have had at least one vaccine dose, 5.7% were Cervarix, 94.3% were Gardasil.

**p < 0.01 between no vaccine and at least one dose.

* p < 0.05 between no vaccine and at least one dose.

*** p < 0.001 between no vaccine and at least one dose.

Table 2 describes respondents’ decision making preferences by vaccination status. The importance of protection against STI's, for self or partner, including HPV, was rated highly by males and females and did not differ between the vaccinated and unvaccinated respondents.

**Table 2:** Decision making preferences by HPV vaccination status.

	No HPV Vaccine	At least one dose of HPV vaccine
	**Mean (SD)**	**Mean (SD)**
Importance† of self-protection from STIs including HPV	96 (14)	96 (14)
Importance† of partner protection from STIs including HPV	94 (19)	93 (18)
	**n (%)**	**n (%)**
Discuss HPV vaccine status prior to new relationship		
*Unlikely or very unlikely	118 (36)	57 (26)
Likely or very likely	164 (49)	107 (48)
*Undecided	50 (15)	58 (26)
Preference for sexual partner to be HPV vaccinated		
***Slightly or no preference	73 (38)	70 (20)
***Strongly prefer or very strongly prefer	41 (21)	215 (60)
***Neutral preference	78 (41)	71 (20)
Would have sex with a person who was…		
Not HPV vaccinated	175 (69)	172 (62)
Not using condoms	77 (31)	104 (38)
Would not have sex with a person who was…		
***Not HPV vaccinated	25 (15)	122 (3)
***Not using condoms	137 (85)	207 (63)
Confidence in self-protection from STI during sex		
Somewhat not or not at all confident	12 (6)	195 (6)
Neutral confidence	15 (7)	18 (6)
Somewhat or very confident	195 (88)	287 (88)
Most important influencer about whether or not to receive the HPV vaccination		
Shared choice between doctor and me	22 (10)	50 (15)
***Me	176 (78)	206 (63)
My doctor	20 (9)	19 (6)
***My parents/guardians	8 (4)	53 (16)
If all doses and boosters were completely free to me, I would get the vaccine		
*Strongly disagree/disagree	32 (14)	28 (8)
**Neutral	68 (30)	62 (19)
***Strongly agree/agree	127 (56)	240 (73)

†Importance scored on a 0–100 Likert scale where 0 was not at all important and 100 was extremely important.

* p < 0.05 between no vaccine doses and at least one dose.

** p < 0.01 between no vaccine doses and at least one dose.

*** p < 0.001 between no vaccine doses and at least one dose.

About 50% of respondents, regardless of vaccination status, said they were likely or very likely to discuss their vaccination status with a new sex partner. On the other hand, significantly more unvaccinated than vaccinated were unlikely or very unlikely to discuss HPV vaccine status prior to a new relationship (36% vs 26%, p < 0.05); and the vaccinated were significantly more ‘undecided’ about the discussion than the unvaccinated (26% vs 15%, p < 0.05).

The vaccinated expressed significantly higher strong or very strong preferences for their sexual partner to be HPV vaccinated compared to the unvaccinated (60% vs 21%, p < 0.001). Conversely, the unvaccinated were neutral or had slight/no preference for partner vaccination significantly more often than the vaccinated (41% vs 20%, p < 0.001; 38% vs 20%, p < 0.001, respectively).

There was no difference between the vaccinated and unvaccinated in preference for preventive measures before having sex with a person: Over half of the vaccinated and unvaccinated would have sex with a person who was not HPV vaccinated; and about a third of the vaccinated and unvaccinated would have sex with a person without using a condom. Asking the negative question about who they would not have sex with, significantly fewer vaccinated would not have sex with a person who did not have HPV vaccination (3% vs 15%, p < 0.001), and similarly, significantly fewer vaccinated would not have sex with a person not using condoms (63% vs. 85%, p < 0.001). Yet, the majority (88%) of respondents indicated a somewhat or very confident stance in preventing self from an STI during sex irrespective of protection by condom use or vaccination.

Respondents were asked who was most important in making the decision to be vaccinated. Options included: “it was a shared choice between the doctor and me”, “myself”, “my doctor”, or “my parents or guardians”. Overall, 69% of respondents chose “myself” as the most important person in the decision whether to vaccinate; the unvaccinated chose “myself” more frequently (78% vs 63%, p < 0.001) than the vaccinated. Overall, 13% indicated that participating in a shared choice was the most important influence to receive vaccination, and this rate was similar between the unvaccinated and vaccinated (10% and 15%, respectively). Although “my parents or guardians” was selected by a small percentage (11% overall), those choosing “my parents or guardians” were more often vaccinated than not (16% vs 4%, p < 0.001). “My doctor” was chosen least often (7% overall) and at a similar rate among the unvaccinated and vaccinated respondents (9% and 6%, respectively).

Finally, overall 65% of the respondents would get vaccinated if all doses and boosters were completely free of charge. For the unvaccinated, 44% remarked that free vaccine would not influence them, whereas for the vaccinated, 73% agreed/strongly agreed that free vaccine would facilitate their vaccination (44% vs. 27%, p < 0.001%, and 56% vs 73%, p < 0.001, respectively).

### 3.1 Knowledge about HPV associated diseases

**Fig. 1:**
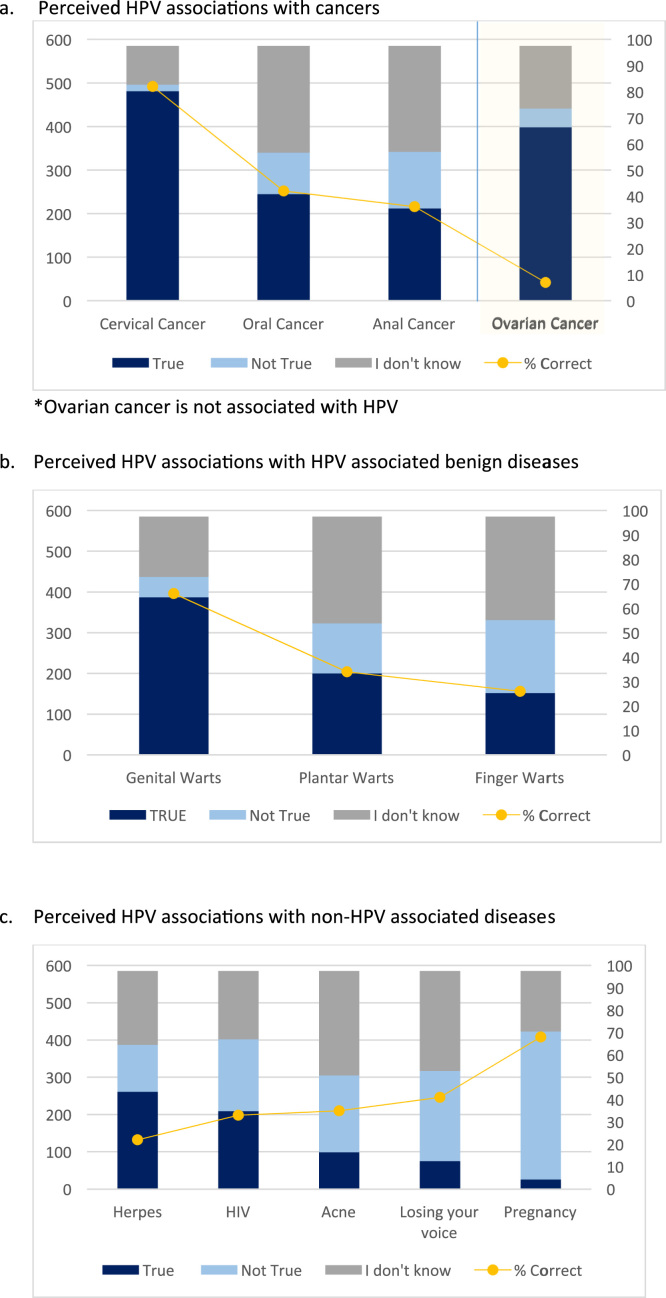
Perceived knowledge of HPV associated diseases. a) Perceived HPV associations with cancers. b) Perceived HPV associations with HPV associated benign diseases. c) Perceived HPV associations with non-HPV associated diseases.

Of the cancers presented to the respondent, cervical cancer most often was correctly attributed to HPV (Fig. 1**a**); ovarian cancer was incorrectly attributed to HPV 93% of the time. Genital warts were correctly attributed to HPV in 66% of the respondents, and other HPV associated benign diseases, such as plantar and finger warts, were correctly identified in 34% and 26% of the respondents, respectively (Fig. 1**b**). There was minimal understanding of the lack of HPV association with other common diseases or conditions, except for pregnancy (Fig. 1**c**).

Out of 12 possible points for correct answers, the median and interquartile ranges for the total *knowledge sub-score for HPV associated diseases* was 5 (3, 7), a score that did not differ by vaccination status, gender, race or age.

### 3.2 Knowledge about HPV vaccines

Overall, respondents had two dominant answers about the duration of vaccine efficacy: 29% responded that the vaccine protected for a lifetime, and 29% indicated that they did not know (Table 3). The vaccinated more often believed the vaccine offered lifetime protection than did the unvaccinated (39% vs. 16%, p < 0.001), and very few understood that there are no data to definitively describe the vaccines’ duration of efficacy.

**Table 3:** Knowledge about HPV and HPV vaccines by vaccination status.

	No vaccine dose	At least one dose
**Knowledge**	n (%)	n (%)
**Duration of vaccine efficacy**		
One year	9 (4)	7 (2)
Up to 5 years	37 (16)	32 (10)
5–10 years	33 (15)	46 (14)
10–20 years	17 (7)	33 (10)
***Lifetime	36 (16)	128 (39)
Science doesn’t know	8 (4)	12 (4)
I don’t know	87 (38)	72 (22)
**Need for cervical cancer screening if you have the HPV vaccine**		
***Definitely yes	131 (58)	235 (71)
Probably yes	74 (33)	82 (25)
I don’t know	21 (9)	11 (3)
Probably not	1 (<1)	2 (1)
Definitely not	0 (0)	0 (0)
**Need for cervical cancer screening if you do NOT have the HPV vaccine**		
***Definitely yes	177 (78)	292 (88)
Probably yes	30 (13)	24 (7)
I don’t know	17 (7)	13 (4)
Probably not	3 (2)	1 (1)
Definitely not	0 (0)	0 (0)
**Finger warts are the same thing as genital warts (caused by the same HPV type)**		
Yes	41 (18)	43 (13)
No	126 (56)	177 (54)
Uncertain	60 (26)	110 (33)

*p < 0.05 between no HPV vaccine and at least one dose.

* *p < 0.01 between no HPV vaccine and at least one dose.

*** p < 0.001 between no HPV vaccine and at least one dose.

Overall, 93% of respondents correctly understood the need for cervical cancer screening with or without HPV vaccination. The vaccinated respondents understood significantly more than the unvaccinated about the need for continued cervical cancer screening regardless of whether vaccination occurred (need for screening if vaccinated: 71% vs. 58%, p < 0.001; if not vaccinated: 88% vs. 78%, p = 0.001).

Out of 3 possible points for correct answers, the median and interquartile ranges for the total *knowledge sub-score for HPV vaccines* was 2 (1, 2). While both groups knew much about HPV vaccines, the unvaccinated knew significantly less about the vaccines than the vaccinated with 88% scoring a 2 or 3 compared to 95% by the vaccinated (p < 0.05).

Summing the *two sub-scores about HPV disease and HPV vaccines together* resulted in a median correct score of 6 with interquartile ranges of 4 and 8 out of a possible 15 total correct. There were no differences between the two groups in knowledge by vaccination status, gender, race or age.

### 3.3 Beliefs about HPV vaccines

Booster shots were acceptable to over 90% of the population (Table 4). Annual boosters were preferred by nearly 50% of the population: significantly more among the vaccinated compared to the unvaccinated (53% vs. 41%, p < 0.01). About a third of the population, regardless of vaccination status, would accept the booster every 5 years. Less than 10% would not accept a booster shot.

**Table 4:** Beliefs about HPV, vaccines, and HPV vaccination.

	No vaccine dose	At least one dose
**Beliefs**	n (%)	n (%)
**Frequency of booster shot acceptance**		
Never	19 (8)	9 (3)
**Yearly	93 (41)	174 (53)
Every 5 years	80 (35)	117 (35)
Every 10 years	35 (15)	30 (9)
***Concern about HPV vaccine safety		
Strongly agree	30 (13)	20 (6)
Agree	57 (25)	67 (20)
Neither agree nor disagree	70 (31)	90 (27)
Disagree	39 (17)	90 (27)
Strongly disagree	31 (14)	63 (19)
**All vaccines, childhood, adolescent and adult, are completely safe**		
Strongly agree	37 (16)	67 (20)
Agree	86 (38)	135 (41)
Neither agree nor disagree	66 (29)	88 (27)
Disagree	29 (13)	35 (11)
Strongly disagree	9 (4)	5 (2)
***If there was no cost to any HPV vaccine or booster, I would get it		
Strongly agree	57 (25)	140 (42)
Agree	70 (31)	100 (30)
Neither agree nor disagree	68 (30)	62 (19)
Disagree	17 (7)	15 (5)
Strongly disagree	15 (7)	13 (4)
**Likelihood of being infected with HPV at some point in your life (0 = certainly no, 100 = certainly yes)**		
Mean (SD)	20.6 (25.5)	15.1 (22.5)
***Median (IQR)	10 (1, 30)	6.5 (0, 20)
**Likelihood of getting cancer if you got a HPV infection (0 = certainly no, 100 = certainly yes)**		
Mean (SD)	33.0 (26.5)	30.4 (28.7)
Median (IQR)	30 (10, 50)	20.5 (4, 50)

*p < 0.05 between no HPV vaccine and at least one dose.

** p < 0.01 between no HPV vaccine and at least one dose.

*** p < 0.001 between no HPV vaccine and at least one dose.

Concerns about HPV vaccine safety were significantly higher among the unvaccinated than the vaccinated (38% vs 26%, p < 0.001), yet over a quarter of the vaccinated agreed or strongly agreed with concerns about HPV vaccine safety. For childhood, adolescent and adult vaccines, in general, over half of all respondents believed they were completely safe, while nearly a third had no opinion.

Most respondents agreed or strongly agreed that free HPV vaccine or booster doses would influence them to get vaccinated; and, those vaccinated were significantly more influenced than the unvaccinated (72% vs. 56%, p < 0.001).

The perception of the likelihood of being infected with HPV was quite low for both groups, but significantly lower for the vaccinated than the unvaccinated (median of 6.5 (IQR 0, 20) vs. 10 (1, 30), p < 0.001). The perception of the likelihood of their HPV infection progressing to cancer did not differ by vaccination status (median 24, IQR 5, 50), but was much higher than the actual risk [9]

### 3.4 Predictors of vaccination status

In a univariate logistic regression analysis (Table 5), all parameters were explored for association with receiving at least one HPV vaccine dose. Age was inversely related to vaccination, as would be expected with national focus on vaccinating pre-adolescents rather than college students. Of note, though, is the lack of significance of the overall knowledge scores for HPV associated diseases and HPV vaccines predicting who would be vaccinated. Likewise, the belief in getting cancer after being HPV infected was not a significant predictor of who would be vaccinated. Finally, the importance of protecting oneself or one's partner from a STI also did not predict vaccination status.

**Table 5:** Univariate Predictors of receiving at least one HPV vaccine dose.

	**OR**	**95% CI**
*Age, yrs	0.85	0.80, 0.90
Knowledge about HPV associated diseases	0.99	0.93, 1.06
Overall knowledge about HPV associated diseases and HPV vaccines	1.00	0.94, 1.07
Belief in having HPV infection during lifetime	0.99	0.98, 0.998
Belief in getting cancer after being HPV infected	1.00	0.99, 1.00
Importance of protecting self from STI	1.00	0.99, 1.01
Importance of protecting partner from STI	1.00	0.99, 1.01
**Gender**		
Female	Referent	
* Male	0.18	0.12, 0.29
**Race**		
White	Referent	
Black	1.50	0.86, 2.63
*Asian	0.40	0.20, 0.82
**Number of lifetime sexual partners**		
0	Referent	
1–2	1.02	0.62, 1.69
3–5	1.22	0.70, 2.11
6–10	1.73	0.91, 3.29
>10	0.74	0.38, 1.43
**Confidence to protect self from STI**		
Not at all confident/somewhat confident	1.32	0.49, 3.57
Neither confident nor not confident	Referent	
Somewhat confident/very confident	1.27	0.60, 2.49
**With new partner, will you discuss whether you each have the HPV vaccine?**		
Very unlikely/unlikely	1.20	0.77, 1.87
Undecided	Referent	
Very likely/likely	1.62	0.99, 2.67
**Preference for partner having HPV vaccine?**		
No preference/slightly prefer	Referent	
Prefer	1.62	0.98, 2.67
*Very strongly/strongly prefer	5.47	3.42, 8.73
**Have sex with someone who has not have HPV vaccination**		
Yes	Referent	
*No	4.97	3.08, 8.02
*Among female respondents only*:		
Yes	Referent	
*No	4.25	2.44, 7.42
*Among male respondents only*:		
Yes	Referent	
*No	6.65	2.32, 19.0
**Have sex with someone who does not use condoms**		
Yes	Referent	
No	1.19	0.78, 1.61
**Who is most important in making a decision about whether or not you get the HPV vaccine?**		
Myself	Referent	
*Shared decision	1.94	1.13, 3.33
Doctor	0.81	0.42, 1.57
*Parents/guardians	5.66	2.62, 12.22
**Knowledge**		
**Knowledge of HPV associated cancers**		
Cervical	1.41	0.91, 2.21
*Anal	0.69	0.49, 0.98
Oropharyngeal	0.78	0.56, 1.10
Ovarian	1.41	0.73, 2.74
**Knowledge of non-HPV associated diseases**		
Herpes	0.92	0.61, 1.39
Acne	1.01	0.71, 1.44
*HIV	0.60	0.42, 0.85
Pregnancy	1.21	0.85, 1.75
Pharyngitis	1.28	0.91, 1.81
**Knowledge of HPV associated benign diseases**		
Finger Warts	1.04	0.72, 1.50
Plantar Warts	1.21	0.84, 1.73
Genital Warts	0.98	0.69, 1.40
**If you are HPV vaccinated, do you still need to have cervical cancer screening?**		
Definitely/probably yes	0.77	0.07, 8.58
I don't know	0.26	0.02, 3.22
Definitely not/probably not	Referent	
**If you are not HPV vaccinated, do you still need to have cervical cancer screening?**		
Definitely/probably yes	4.58	0.47, 44.33
I don't know	2.29	0.21, 24.68
Definitely not/probably not	Referent	


**Beliefs**		
**How often would you be willing to receive a HPV booster shot, if one was recommended?**		
Never	Referent	
**Yearly	3.95	1.72, 9.01
**Every 5 years	3.09	1.33, 7.17
Every 10 years	1.81	0.72, 4.59
*Among female respondents only:*		
Never	Referent	
**Yearly	4.46	1.82, 10.91
**Every 5 years	3.02	1.23, 7.44
Every 10 years	2.33	0.83, 6.52
**Duration of vaccine protection**		
One year	0.52	0.14, 1.97
Up to 5 years	0.58	0.21, 1.59
5–10 years	0.93	0.34, 2.53
10–20 years	1.29	0.44, 3.77
Up to a lifetime	2.37	0.90, 6.24
Science does not know	Referent	
**I am concerned with HPV vaccine safety**		
**Strongly/very strongly disagree	1.70	1.12, 2.59
Neither agree/disagree	Referent	
Agree/ strongly agree	0.78	0.51, 1.20
**All vaccines are safe**		
Strongly/very strongly disagree	0.79	0.46, 1.36
Neither agree/disagree	Referent	
Agree/ strongly agree	1.23	0.83, 1.82
**Influence of free HPV vaccine/boosters**		
Strongly/disagree	0.96	0.52, 1.77
Neither agree/disagree	Referent	
**Strongly/agree	2.07	1.38, 3.11

* Indicates statistical significance.

** Indicates statistical significance.

Men were significantly less likely to be vaccinated than women (OR = 0.18, 95% CI: 0.12, 0.29), and those declaring Asian race/ethnicity were less likely to be vaccinated than whites (OR = 0.40, 95% CI: 0.20, 0.82). Number of lifetime sexual partners was not predictive of vaccination status, nor was confidence about protecting self from STIs.

While the discussion with new sex partners about each having been vaccinated was not predictive of vaccination status, vaccinated respondents indicated a very strong preference for their partner to have been vaccinated compared to the unvaccinated respondent (OR=5.47, 95% CI: 3,42, 8.73). This was reinforced by the vaccinated men and women stating that they were significantly more likely to not have sex with someone who has not had the HPV vaccine (OR _males only_ = 6.65, 95% CI: 2.32, 19.00, OR _females only_ = 4.25 95% CI: 2.44, 7.42). On the other hand, vaccinated respondents compared to the unvaccinated were not more likely to care whether their partner used condoms.

Respondents, vaccinated or not, rarely indicated the doctor was most important influence on whether to get the HPV vaccine. The endorsement of the options, “a shared choice” or “my parents or guardians” was predictive of being vaccinated (OR = 1.94, 95% CI: 1.13, 3.33, OR=5.66 95% CI: 2.62, 12.22, respectively).

Among knowledge questions, the vaccinated were significantly more likely to be unaware of the association of HPV with anal cancer (OR = 0.69, 95% CI: 0.49, 0.98). In addition, the vaccinated were also less aware of the lack of association between HPV and HIV (OR = 0.60, 95% CI: 0.42, 0.83) than the unvaccinated. HPV associated benign disease knowledge did not predict vaccine uptake.

Understanding the need for continued cervical cancer screening did not significantly predict vaccination status.

Belief in yearly or every 5 year booster doses was significantly associated with vaccine uptake, especially among the female responders (OR = 4.46, 95% CI: 1.82, 10.91 and OR = 3.02, 95% 1.23, 7.44, respectively). However, the knowledge about the duration of vaccine efficacy was not a significant predictor of vaccine uptake.

The vaccinated respondents were significantly more likely than the unvaccinated to strongly/very strongly disagree with concerns about HPV vaccine safety (OR = 1.70, 95% CI: 1.12, 2.59), while at the same time having no difference from their unvaccinated respondents in agreement about the safety of childhood and adult vaccines. Finally, the influence of free vaccine/booster on vaccine uptake was significant (OR = 2.07, 95% CI: 1.38, 3.11).

### 3.5 Multivariate predictors of HPV vaccination

The multivariate analyses considered all of the significant univariate predictors in an adjusted model (Table 6). Older age, male gender and Asian ethnicity all predicted less people vaccinated as they aged compared to younger ages, females, or the white race, respectively in this adjusted model.

**Table 6:** Multivariate analysis of predictors of vaccination.

	aOR	95% CI
*Age, yrs	0.83	0.76, 0.90
**Gender**		
*Male	0.13	0.07, 0.24
Female	Referent	
**Race/ethnicity**		
White	Referent	
Black	1.67	0.80, 3.51
*Asian	0.14	0.06, 0.37
**Correct knowledge about HPV diseases and HPV vaccines**		
Cervical cancer	0.66	0.33, 1.35
Oral cancer	1.51	0.85, 2.68
Anal cancer	1.67	0.99, 2.81
Ovarian cancer	0.38	0.14, 1.03
Genital Warts	1.29	0.73, 2.27
*Plantar Warts	0.47	0.26, 0.87
Finger Warts	0.81	0.45, 1.48
*Finger/Genital Wart	2.05	1.08, 3.91
Herpes	0.68	0.34, 1.34
Acne	1.05	0.60, 1.86
HIV	1.28	0.70, 2.35
Pregnancy	0.68	0.37, 1.25
Pharyngitis (loss of voice)	1.06	0.60, 1.90
Duration of Vaccination Efficacy	1.41	0.41, 4.78
**Frequency for booster vaccines, if needed**		
Never	Referent	
Yearly	1.59	0.48,5.26
Every 5 years	1.26	0.38, 4.25
Every 10 years	1.03	0.28, 3.76
**Preference for partner to be HPV vaccinated**		
*No preference/slightly prefer	0.50	0.27, 0.93
Prefer	Referent	
*Very strongly/strongly prefer	4.04	2.31, 7.05
**Important influencers in decision to be vaccinated or not**		
Myself	Referent	
Shared choice	1.97	0.99, 3.92
Doctor	1.14	0.48, 2.71
*Parent/Guardian	5.32	2.17, 13.03
**Concern about HPV vaccine safety**		
Strongly/very strongly disagree	0.64	0.29, 1.39
Neither agree/disagree	Referent	
Agree/strongly agree	1.15	0.66, 2.01
**Influence of free vaccine/booster**		
Strongly/very strongly disagree	1.28	0.52, 3.11
Neither agree/disagree	Referent	
*Agree/strongly agree	1.90	1.05, 3.41

Adjustments made for all variables listed for adjusted odds ratio calculations.

* Indicates statistical significance.

Several other important results from this analysis also appeared. Lack of concern about HPV vaccine safety did not influence vaccine uptake in the adjusted model, neither did the need for boosters at any frequency. Knowledge in limited areas did predict vaccine uptake, though. Specifically, those who correctly answered that finger and genital warts were from different HPV types were twice as likely to be HPV vaccinated (aOR 2.05 (1.08, 3.91)); and those who correctly categorized plantar warts as caused by HPV were significantly less likely to be vaccinated.

Being vaccinated was predicted by having a very strong/strong preference for the partner to also be vaccinated (aOR=4.04, 95% CI: 2.31, 7.05); while having no or little preference for partner vaccination was significantly associated with less vaccine uptake.

Significant influencers to be vaccinated were reduced to only parent/guardian influence in the multivariate model (aOR 5.32 (2.17, 13.03) with the opportunity for shared choice or the doctor's recommendation becoming not significant. Finally, free vaccine/boosters predicted an increased vaccine uptake by nearly two-fold (aOR 1.90 (1.05, 3.41).

## 4 Discussion

This study is one of the larger studies to date to investigate the knowledge about HPV associated diseases and vaccines, attitudes and beliefs in college students since the implementation of the Patient Protection and Affordable Care Act (ACA) in 2010. The ACA makes it more likely that college aged adults can be vaccinated, regardless of ability to pay. Kentucky has had a significant Medicaid expansion through the ACA and has reduced the uninsured rates to less than 11% [10].

In 2014, the most recent report, 12.5% of females were first vaccinated at 18 years of age and 20% at 19 years or older; among males, 24.4% were first vaccinated at 18 years of age and 26% at 19 years or older, despite the target age for vaccination being 11–12 years old [2]. The older ages at which HPV vaccines are accepted and received, separate from the pre-pubescent age, is important to acknowledge, as nearly half of females and males are vaccinated as adults [2]. Adding young adult HPV vaccination programs to ongoing cancer prevention outreach can only be helpful in reducing HPV infections and their cancerous sequelae [11]. Hence, efforts to improve catch up vaccination among young adults, attending college or not, are urgently needed.

The young adults attending college in urban Kentucky have a higher rate of vaccination than is reported for the nation, but similar to the reports from the American College of Health Association [4]. We show that negative predictors of HPV vaccine uptake are increasing age, male gender and Asian race, all descriptors previously shown in other work [12]. In this work we have shown that knowledge and beliefs about HPV infection and associated cancers are quite low and are not predictors of HPV vaccine uptake, as has been reported in other college aged surveys [6], [7]. This stands with two unique exceptions: those who could correctly discriminate between finger and genital wart HPV types had a significantly likelihood of vaccine uptake; but, the inability to link plantar warts with HPV infection, albeit different HPV types than genital warts or cancers, was associated with a negative predictor for vaccine uptake.

The concept of free vaccine was associated with vaccine uptake in this college-aged survey, but real life free vaccine has not been significant for an increased uptake or compliance with HPV4 vaccine in a different young adult population [13]. Likewise, the concept of booster vaccines at any interval was not a predictor of vaccine uptake.

One of the most important outcomes of this study was the finding that a very strong/strong preferences for their sexual partner to be vaccinated was a highly significant predictor of HPV vaccine uptake. This result is concordant with other work supporting the female's desire for her male partner to be HPV vaccinated [14]. This projection of health benefit due to a partner's actions rather than taking one's own responsibility is a familiar health conundrum also seen most often around the contraception discussion [15]. Future behavioral communication and psychology work in this area is needed.

Specifically, but contrary to many adolescent focused studies [16], [17] and one young adult study [18], a doctor's recommendation, alone, had the least influence in vaccine uptake for these college aged students. While our respondents overwhelmingly claimed the decision as their own (69%), this self-determination did not result in increased vaccine uptake, rather equal proportions accepted and rejected the HPV vaccination. The influence of a shared choice (13%) was small in magnitude, and lost significance when partner preferences were considered.

Parental influence (11%) was also a significant predictor of vaccine uptake as has been discussed in other similar communities [19], [20], [21]. Positive parenting has been shown to reverse or negate the effects of peer influence/neighborhood influence in late adolescence [22], potentially indicating a possible success in renewed effort at directing health training and education to all adults who act in a parenting role. Often parenting occurs at many levels of familial structure, supporting the need for lay articles about HPV associated cancer prevention in many age-directed media outlets.

Finally, we found little evidence that students were overly concerned with vaccine safety, especially among those who were vaccinated. This finding is consistent with a previous systematic review [23].

## 5 Conclusions

Increasing young adult vaccination rates may come with changing preferences and influencers. Finding mechanisms to move young adults to prefer partner vaccination may offer an increase in HPV vaccine uptake, similar to peer pressure or peer education for contraceptive choices. In general, to observe population effects in cervical cancer prevention, we must include post adolescent young adults in the vaccination drive. Co-messaging HPV vaccination and cervical cancer screening to young adults may be the best approach for reducing cervical cancer burden later in life [24]. Lastly, educating young adults on how to navigate difficult discussions about condom usage and other STI protection methods may lead to increased frank communication and more confidence in protecting one's sexual health [25].

### 5.1 Limitations

We did not address the number of doses that the young adults completed, nor the time frame in which they might have received more than one dose. With two dose regimens being promoted by the WHO [26] and one dose regimens being trialed [Clinicaltrials.gov number NCT03180034], the behavioral health attitudes of reduced numbers of dosing in young adults may influence uptake that is not considered in this work.

This is a survey conducted from a convenience population sample. The survey was designed to incorporate self-validation for maximizing truthful responses with control questions, options to not answer a question, anonymity, and no signed consent form. Generalizability to other college aged populations cannot be verified. Respondents were all enrolled in college and, at minimum, had access to campus health services; these results, therefore, may not apply to young adults not in college, or those with limited access to health care. Future research among the vocational young adults is needed to understand how to reach others not in a collegiate setting. Additional studies are needed to assess how to teach strategies to young adults so that they can inform new partners of the expected protections (vaccination, condoms, other prophylactic measures) the young adult has of a new partner.

## Conflict of interest

No author has any conflict of interest for publishing this work.

## Funding source

This research did not receive any specific grant from funding agencies in the public, commercial, or not for profit sectors.

## References

[1] Markowitz L.E., Dunne E.F., Saraiya M., Chesson H.W., Curtis C.R., Gee J., Bocchini J.A., Unger E.R. (2014). Centers for disease control and prevention (CDC). human papillomavirus vaccination: recommendations of the Advisory Committee on immunization practices (ACIP). MMWR Recomm. Rep..

[2] Williams W.W., Lu P.J., O'Halloran A., Kim D.K., Grohskopf L.A., Pilishvili T., Skoff T.H., Nelson N.P., Harpaz R., Markowitz L.E., Rodriguez-Lainz A., Fiebelkorn A.P. (2017). urveillance of vaccination coverage among adult populations - United States, 2015. MMWR Surveill. Summ..

[3] Thompson E.L., Vamos C.A., Vázquez-Otero C., Logan R., Griner S., Daley E.M. (2016). Trends and predictors of HPV vaccination among U.S. College women and men. Prev. Med..

[4] Kuo P.F., Yeh Y.T., Sheu S.J., Wang T.F. (2014). Factors associated with future commitment and past history of human papilloma virus vaccination among female college students in northern Taiwan. J. Gynecol. Oncol..

[5] McBride K.R., Singh S. (2017). Predictors of adults' knowledge and awareness of HPV, HPV-associated cancers, and the HPV vaccine: implications for health education. Health Educ. Behav..

[6] Wolwa M., Blavo C., Shah R., Fleisher J.M., Espinal T. (2013). Cervical cancer knowledge and prevention among college women. J. Community Health.

[7] M. Oz, N. Cetinkaya, A. Apaydin, E. Korkmaz, S. Bas, E. Ozgu, T. Gungor, Awareness and Knowledge Levels of Turkish College Students about Human Papilloma Virus Infection and Vaccine Acceptance. J Cancer Educ Sep 21, 2016. 10.1007/s13187-016-1116-0 27655177

[8] Alumran A., Hou X.-Y., Hurst C. (2012). Validity and reliability of instruments designed to measure factors influencing the overuse of antibiotics. J. Infect. Public Health.

[9] Schiffman M., Rodríguez A.C. (2008). Heterogeneity in CIN3 diagnosis. Lancet Oncol..

[10] Benitez J.A., Creel L., Jennings J. (2016). Kentucky's medicaid expansion showing early promise on coverage and access to care. Health Aff..

[11] Burger E.A., Sy S., Nygård M., Kristiansen I.S., Kim J.J. (2015). Too late to vaccinate? The incremental benefits and cost-effectiveness of a delayed catch-up program using the 4-valent human papillomavirus vaccine in Norway. J. Infect. Dis..

[12] Jeudin P., Liveright E., Del Carmen M.G., Perkins R.B. (2014). Race, ethnicity, and income factors impacting human papillomavirus vaccination rates. Clin. Ther..

[13] Harper D.M., Verdenius I., Harris G.D., Barnett A.L., Rosemergey B.E., Arey A.M., Wall J., Malnar G.J. (2014). The influence of free quadrivalent human papillomavirus vaccine (HPV4) on the timely completion of the three dose series. Prev. Med..

[14] Harper D.M., Alexander N.M., Ahern D.A., Comes J.C., Smith M.S., Heutinck M.A., Handley S.M. (2014). Women have a preference for their male partner to be HPV vaccinated. PLoS One.

[15] 〈http://www.nytimes.com/1995/05/23/us/study-finds-little-male-responsibility-in-birth-control.html〉. (Accessed July 4, 2017).

[16] Sturm L., Donahue K., Kasting M., Kulkarni A., Brewer N.T., Zimet G.D. (2017). Pediatrician-parent conversations about human papillomavirus vaccination: an analysis of audio recordings. J. Adolesc. Health.

[17] Hswen Y., Gilkey M.B., Rimer B.K., Brewer N.T. (2017). Improving physician recommendations for human papillomavirus vaccination: the role of professional organizations. Sex. Transm. Dis..

[18] Pierre Joseph N., Clark J.A., Mercilus G., Wilbur M.A.B., Figaro J., Perkins J. (2014). Racial and ethnic differences in HPV knowledge, attitudes, and vaccination rates among low-income African-American, Haitian, Latina and Caucasian young adult women. J. Pediatr. Adolesc. Gynecol..

[19] Krawczyk A., Perez S., King L., Vivion M., Dubé E., Rosberger Z. (2015). Parents' decision-making about the human papillomavirus vaccine for their daughters: II. Qualitative results. Hum. Vaccin Immunother..

[20] Krawczyk A., Knäuper B., Gilca V., Dubé E., Perez S., Joyal-Desmarais K., Rosberger Z. (2015). Parents' decision-making about the human papillomavirus vaccine for their daughters: I. Quantitative results. Hum. Vaccin Immunother..

[21] Casey B.R., Crosby R.A., Vanderpool R.C., Dignan M., Bates W. (2013). Predictors of initial uptake of human papillomavirus vaccine uptake among rural Appalachian young women. J. Prim. Prev..

[22] Whittle S., Vijayakumar N., Simmons J.G., Dennison M., Schwartz O., Pantelis C., Sheeber L., Byrne M.L., Allen N.B. (2017). Role of positive parenting in the association between neighborhood social disadvantage and brain development across adolescence. JAMA Psychiatry.

[23] Holman D.M., Bernard V., Roland K.B., Watson M., Liddon N., Stokely S. (2014). Barriers to human papillomavirus vaccination among US adolescents. A systematic review of the literature. JAMA Pediatr..

[24] Bosch F.X., Robles C., Díaz M., Arbyn M., Baussano I., Clavel C., Ronco G., Dillner J., Lehtinen M., Petry K.U., Poljak M., Kjaer S.K., Meijer C.J., Garland S.M., Salmerón J., Castellsagué X., Bruni L., de Sanjosé S., Cuzick J. (2016). HPV-FASTER: broadening the scope for prevention of HPV-related cancer. Nat. Rev. Clin. Oncol..

[25] Tulloch H.E., McCaul K.D., Miltenberger R.G., Smyth J.M. (2004). Partner communication skills and condom use among college couples. J. Am. Coll. Health.

[26] 〈http://www.who.int/immunization/diseases/hpv/en/〉 (Accessed 20 January 2018).

